# PD-L1 expression in tonsillar cancer is associated with human papillomavirus positivity and improved survival: implications for anti-PD1 clinical trials

**DOI:** 10.18632/oncotarget.12776

**Published:** 2016-10-20

**Authors:** Angela M Hong, Ricardo E Vilain, Sarah Romanes, Jean Yang, Elizabeth Smith, Deanna Jones, Richard A Scolyer, C Soon Lee, Mei Zhang, Barbara Rose

**Affiliations:** ^1^ Sydney Medical School, The University of Sydney, NSW, Australia; ^2^ Tissue Pathology and Diagnostic Oncology, Royal Prince Alfred Hospital, NSW, Australia; ^3^ Pathology North (Hunter), John Hunter Hospital, Newcastle, NSW, Australia; ^4^ School of Mathematics and Statistics, The University of Sydney, NSW, Australia; ^5^ Discipline of Pathology, School of Medicine, Western Sydney University, NSW, Australia

**Keywords:** tonsillar cancer, oropharyngeal cancer, human papillomavirus, PD-L1, p16

## Abstract

In this study, we examined PD-L1 expression by immunohistochemistry in 99 patients with tonsillar cancer and known human papillomavirus (HPV) status to assess its clinical significance. We showed that the pattern of PD-L1 expression is strongly related to HPV status. The PD-L1 positivity rate was 83.3% in HPV-positive cases and 56.9% in HPV-negative cases (*p* < 0.05). Patients with HPV-positive/PD-L1-positive cancer had significantly better event free survival and overall survival compared with patients with HPV-negative/PD-L1-negative cancer. Relative to those patients with HPV-negative/PD-L1-negative disease who had the highest risk of death, patients with HPV-positive/PD-L1-positive cancers had a 2.85 fold lower risk of developing an event (HR 0.35, 95% CI: 0.16–0.79) and a 4.5 fold lower risk of death (HR =0.22, 95% CI: 0.09–0.53). Our findings will help to guide future clinical trial design in immunotherapy based on PD-L1 expression in tonsillar cancer.

## INTRODUCTION

Oropharyngeal squamous cell carcinoma (OSCC) is a clinically and biologically heterogeneous disease. Human papillomavirus (HPV) is implicated in the majority (up to 70%) of OSCC in the western world with the tonsillar subsite associated with the highest HPV positivity rate [1, 2]. HPV+positive OSCC demonstrates favorable prognosis due to improved response to therapy [3–7]. In contrast, patients with HPV-negative OSCC tend to have a smoking history [8] and to have a variable but poorer prognosis than patients with HPV-positive tumors. Numerous studies have investigated the clinical usefulness of other markers of prognosis in both HPV-positive and HPV-negative OSCC [9, 10].

Programmed Death Receptor 1 (PD1) is one of the members of the extended family of T-cell regulators expressed on the surface of activated T-cells, B-cells and macrophages [11]. Its ligand, Programmed Death Receptor Ligand 1 (PD-L1), is a cell-surface protein that is expressed on cancer cells, macrophages, T-cells and other tissues. The interaction of PD1 and PD-L1 can suppress the cytotoxic CD8 T-cell mediated immune response. There has been immense interest in anti-PD1/anti-PD-L1 immunotherapy in cancer, including head and neck cancer [12]. In a dose escalation study, PD-L1 expression has been described as a predictive marker for response to anti-PD1 immunotherapy in melanoma, lung cancer, renal cell cancer, colorectal cancer and prostate cancer[13].

In this study, we analysed PD-L1 expression by immunohistochemistry in a well-defined cohort of tonsillar cancer with known HPV status to assess its clinical significance.

## RESULTS

The baseline characteristics and PD-L1 positivity of the study cohort by HPV status are shown in Table 1. Forty-eight of 99 patients (48.5%) had HPV-positive disease (HPV DNA-positive, p16-positive). Patients with HPV-positive tumors were significantly younger at diagnosis, more likely to be never smokers and non-drinkers, and had higher tumor grade, higher N-stage and higher overall tumors stage compared to patients with HPV-negative tumors. HPV-positive tumors were strongly associated with PD-L1 positivity (83.3% vs 56.9%, *p* < 0.05). Within the HPV-positive group, there was no significant difference in PD-L1 positivity by smoking status (never smoker 87.5%, ex and current smoker 79.2%, *p* = 0.249). The number of never smokers within the HPV-negative group was too small for meaningful statistical analysis. In terms of the presence of TILs within the primary tumors, there was no difference between HPV-positive cancer and HPV-negative cancer. However, there was a statistically significant difference in the pattern of TIL distribution, with HPV-positive cancer more likely to have > 25% TIL distribution within the tumor.

**Table 1:** Demographic and clinical characteristics of the study population by HPV status

	All Patients (*N* = 99)	HPV-Positive (*N* = 48, 48.5%)	HPV-Negative (*N* = 51, 51.5%)	*P*-value
Median age at diagnosis (range)	58 (34–83)	57.5 (34–81)	61 (44–83)	0.0029
Gender				
Female	20 (20.2%)	10 (20.8%)	10 (19.6%)	1.00
Male	79 (79.8%)	38 (79.2%)	41 (80.4%)	
Smoking status at diagnosis				
Never smoker	27 (27.3%)	23 (47.9%)	4 (7.8%)	0.0001
Ex-smoker	30 (30.3%)	16 (33.3%)	14 (27.5%)	
Current smoker	42 (42.4%)	9 (18.8%)	33 (64.7%)	
Alcohol status (Missing = 3)				
Non-drinker	12 (12.5%)	9 (18.8%)	3 (6.3%)	0.0001
Ex-drinker	7 (7.3%)	4 (8.3%)	3 (6.3%)	
Current drinker	77 (80.2%)	35 (72.9%)	42 (87.5%)	
Grade				
1	7 (7.1%)	3 (6.3%)	4 (7.8%)	0.0001
2	51 (51.5%)	16 (33.3%)	35 (68.6%)	
3	41 (41.4%)	29 (60.4%)	12 (23.5%)	
T stage				
1	16 (16.2%)	9 (18.8%)	7 (13.7%)	0.0227
2	40 (40.2%)	22 (45.8%)	18 (35.3%)	
3	28 (28.3%)	11 (22.9%)	17 (33.3%)	
4	15 (15.2%)	6 (12.5%)	9 (17.6%)	
N stage				
0	33 (33.3%)	12 (25.0%)	21 (41.2%)	0.0001
1	23 (23.2%)	10 (20.8%)	13 (25.5%)	
2	37 (37.4%)	22 (45.8%)	15 (29.4%)	
3	6 (6.1%)	4 (8.3%)	2 (3.9%)	
TNM Stage				
1	4 (4.0%)	0	4 (7.8%)	0.0001
2	17 (17.2%)	8 (16.7%)	9 (17.6%)	
3	26 (26.3%)	10 (20.8%)	16 (31.4%)	
4	52 (52.5%)	30 (62.5%)	22 (43.1%)	
Treatment				
Definitive Radiotherapy +/− Chemo	35 (35.4%)	16 (33.3%)	19 (37.3%)	0.0188
Surgery + Adjuvant Radiotherapy +/−Chemo	54 (54.5%)	29 (60.4%)	25 (49.0%)	
Surgery alone	10 (10.1%)	3 (6.3%)	7 (13.7%)	
PD-L1 Status				
Positive	69 (69.7%)	40 (83.3%)	29 (56.9%)	0.008
Negative	30 (30.35)	8 (16.7%)	22 (43.1%)	
Tumor infiltrating lymphocytes (TIL)				
Yes	85 (85.9%)	43 (89.6%)	42 (82.4%)	0.391
No	14 (14.1%)	5 (10.4%)	9 (17.6%)	
TIL Distribution (1 = < 25%, 2 = 25- < 75%, 3 => 75%)				
− 0 (no TILs)	14 (14.1%)	5 (10.4%)	9 (17.7%)	0.0004
− 1	37 (37.4%)	15 (31.3%)	22 (43.1%)	
− 2	38 (38.4%)	22 (45.8%)	16 (31.4%)	
− 3	10 (10.1%)	6 (12.5%)	4 (7.8%)	

There was no significant difference in age and gender when the study cohort was classified by PD-L1 status (Table 2). Patients with PD-L1 positive tonsillar cancer were more likely to be never smokers and non-drinkers. They were also more likely to present with grade 3 disease, lower T stage and higher N stage disease.

**Table 2:** Demographic and clinical characteristics of the study population by PD-L1 status

	All Patients (*n* = 99)	PD-L1 Positive (*n* = 69)	PD-L1 Negative (*n* = 30)	*P* value
Median age	58 (range 34–83)	58 (range 34–83)	59 (range 38–82)	
Gender				
F	20 (20.2%)	14 (20.3%)	6 (20.0%)	1.00
M	79 (79.8%)	55 (79.7%)	24 (80.0%)	
Smoking status				
Never smoker	27 (27.3%)	24 (34.8%)	3 (10.0%)	0.0001
Ex-smoker	30 (30.3%)	21 (30.4%)	9 (30.0%)	
Current smoker	42 (42.4%)	24 (34.8%)	18 (60.0%)	
Alcohol status (Missing = 3)				
Non-drinker	12 (12.5%)	11 (16.2%)	1 (3.6%)	0.0001
Ex-drinker	7 (7.3%)	4 (5.9%)	3 (10.7%)	
Current drinker	77 (80.2%)	53 (77.9%)	24 (85.7%)	
Grade				
1	7 (7.1%)	5 (7.2%)	2 (6.7%)	0.0011
2	51 (51.5%)	32 (46.4%)	19 (63.3%)	
3	41 (41.4%)	32 (46.4%)	9 (30.0%)	
T stage				
1	16 (16.2%)	12 (17.4%)	4 (13.3%)	0.0001
2	40 (40.2%)	33 (47.8%)	7 (23.3%)	
3	28 (28.3%)	17 (24.6%)	11 (36.7%)	
4	15 (15.2%)	7 (10.1%)	8 (26.7%)	
N stage				
0	33 (33.3%)	20 (29.0%)	13 (43.3%)	0.0001
1	23 (23.2%)	14 (20.3%)	9 (30.0%)	
2	37 (37.4%)	29 (42.0%)	8 (26.7%)	
3	6 (6.1%)	6 (8.7%)	0	
TNM Stage				
1	4 (4.0%)	2 (2.9%)	2 (6.7%)	0.024
2	17 (17.2%)	13 (18.8%)	4 (13.3%)	
3	26 (26.3%)	16 (23.2%)	10 (33.3%)	
4	52 (52.5%)	38 (55.1%)	14 (46.7%)	
Treatment				
Radiotherapy + Chemo	27 (27.3%)	15 (21.7%)	12 (40.0%)	0.001
Radiotherapy Alone	8 (8.1%)	3 (4.3%)	5 (16.7%)	
Surgery + Radiotherapy +/−Chemo	54 (54.5%)	46 (66.7%)	8 (26.7%)	
Surgery +/− Chemo	10 (10.1%)	5 (7.2%)	5 (16.7%)	

### Outcome analysis

The median follow up time was 56 months (range 1–184 months). A total of 31 patients (31.3%) developed a recurrence. Locoregional recurrence occurred in 25 patients, with failure occurring only at the primary site in 15 patients and only at the regional nodal area in 10 patients. Seven patients developed distant metastases as the first site of recurrence, none of whom had loco-regional failure at the time of the diagnosis of distant metastases. There were 46 events and 38 deaths from any cause, of which 25 patients died from the tonsillar cancer while 13 died from unrelated causes.

Univariate associations of patient and disease characteristics with locoregional failure, any event and death from any cause are shown in Table 3. There was no factor that significantly predicted the presence of locoregional recurrence. Gender, T stage, HPV status and PD-L1 status were prognostic factors of event-free survival. Gender, smoking status, T stage, HPV status and PD-L1 status were all significant prognostic factors for overall survival.

**Table 3:** Univariate associations of patient and disease characteristics with outcome in all patients

Characteristic	Locoregional Failure (*n* = 25)		Any event (*n* = 46)		Death from any cause (*n* = 38)	
	HR (95% CI)	*P*-value	HR (95% CI)	*P*-value	HR (95% CI)	*P*-value
Age at diagnosis						
< 60	0.54 (0.23, 1.28)	0.162	1.08 (0.60, 1.95)	0.79	0.99 (0.52, 1.89)	0.975
>= 60	1	−	1	−	1	−
Gender						
Male			4.19 (1.30, 13.53)	0.0165	5.10 (1.22, 21.22)	0.025
Female	−	−	1	−	1	−
Smoking Status						
Never Smoker	1	−	1	−	1	−
Current/Ex-smoker	2.04 (0.69, 6.06)	0.197	1.63 (0.78, 3.39)	0.191	3.67 (1.30, 10.37)	0.0142
T-Stage						
T1	1		1	−	1	−
T2	0.92 (0.23, 3.73)	0.904	0.93 (0.35, 2.41)	0.853	0.73 (0.24, 2.20)	0.576
T3	1.30 (0.33, 5.16)	0.711	1.67(0.65, 4.32)	0.286	1.70 (0.60, 4.78)	0.316
T4	1.67 (0.39, 7.08)	0.485	2.99 (1.10, 8.14)	0.032	3.46 (1.17, 10.21)	0.025
N-Stage						
N0	1	−	1	−	1	−
N1	0.56 (0.19, 1.64)	0.293	1.21 (0.56, 2.67)	0.642	0.97 (0.41, 2.32)	0.952
N2	0.35 (0.1, 1.08)	0.068	1.03 (0.51, 2.09)	0.939	0.932 (0.43, 2.02)	0.858
N3	0.88 (0.10, 7.52)	0.908	1.69 (0.48, 5.90)	0.412	1.06 (0.24, 4.72)	0.943
Grade						
1			1.40 (0.50, 3.9)	0.522	1.901 (0.68, 5.40)	0.224
2, 3	−	−	1	−	1	−
HPV status						
Positive	0.72 (0.30, 1.76)	0.475	0.48 (0.26, 0.89)	0.019	0.33 (0.16, 0.68)	0.003
Negative	1	−	1	−	1	−
PD-L1 status						
Positive	0.56 (0.24, 1.30)	0.178	0.54 (0.23, 0.99)	0.045	0.46 (0.24, 0.88)	0.019
Negative	1	−	1	−	1	−

In the multivariate analysis (Table 4), there was no significant factor associated with locoregional recurrence. Gender and HPV status were significant prognostic factors for event-free and overall survival. Males were more likely to have an event (HR 6.67, 95% CI 1.80–24.78) and to die (HR 5.22, 95% CI 1.20–24.40). Patients with HPV-positive cancer were less likely to develop an event (HR 0.37, 95% CI 0.15–0.87) and less likely to die (HR 0.39, 95% CI 0.15–0.99) despite presenting at a more locally advanced stage of disease than patients with HPV-negative cancer. PD-L1 status was not a prognostic factor for survival after adjusting for other variables, including treatment.

**Table 4:** Multivariate associations of patient and disease characteristics with outcome in all patients

Characteristic	Locoregional Failure (*n* = 25)		Any event (*n* = 46)		Death from any cause (*n* = 38)	
	HR (95% CI)	*P*-value	HR (95% CI)	*P*-value	HR (95% CI)	*P*-value
Age at diagnosis						
< 60	0.38 (0.09, 1.53)	0.172	0.66 (0.34, 1.44)	0.330	0.62 (0.28, 1.40)	0.250
>= 60	1	−	1	−	1	−
Gender	N/A					
Male			6.67 (1.80, 24.78)	0.005	5.22 (1.20, 24.40)	0.036
Female			1	−	1	−
T-Stage						
T1	1	−	1	−	1	−
T2	0.57 (0.06, 5.09)	0.614	0.85 (0.29, 2.47)	0.764	0.66 (0.20, 2.21)	0.501
T3	1.32(0.22, 8.07)	0.761	1.14(0.40, 3.25)	0.808	1.02 (0.33, 3.21)	0.966
T4	1.37 (0.13, 14.53)	0.794	2.11 (0.67, 6.64)	0.201	2.73 (0.79, 9.43)	0.113
N-Stage						
N0	1	−	1	−	1	−
N1	0.31 (0.06, 1.69)	0.177	1.26 (0.55, 2.93)	0.584	0.92(0.36, 2.34)	0.856
N2	0.174 (0.03, 1.10)	0.063	1.4085 (0.5966, 3.3256)	0.435	1.0652 (0.4266, 2.6598)	0.892
N3	2.0847 (0.20152, 21.567)	0.538	3.85 (0.91, 16.32)	0.068	2.56(0.48, 13.77)	0.273
Grade	N/A					
1			1.28 (0.41, 3.96)	0.671	1.59 (0.49, 5.12)	0.439
2, 3			1	−	1	−
Smoking Status						
Never Smoker	1	−	1	−	1	−
Current/Ex-smoker	0.36 (0.35, 21.78)	0.339	0.43 (0.15, 1.20)	0.106	1.08 (0.30, 3.93)	0.906
HPV status						
Positive	1.53 (0.30, 7.86)	0.609	0.37 (0.15, 0.87)	0.024	0.39 (0.15, 0.99)	0.050
Negative	1	−	1	−	1	−
PD-L1 status						
Positive	0.54 (0.16, 1.89)	0.337	0.59 (0.29 1.18)	0.134	0.66 (0.31, 1.37)	0.261
Negative	1	−	1	−	1	−

**Figure 1:**
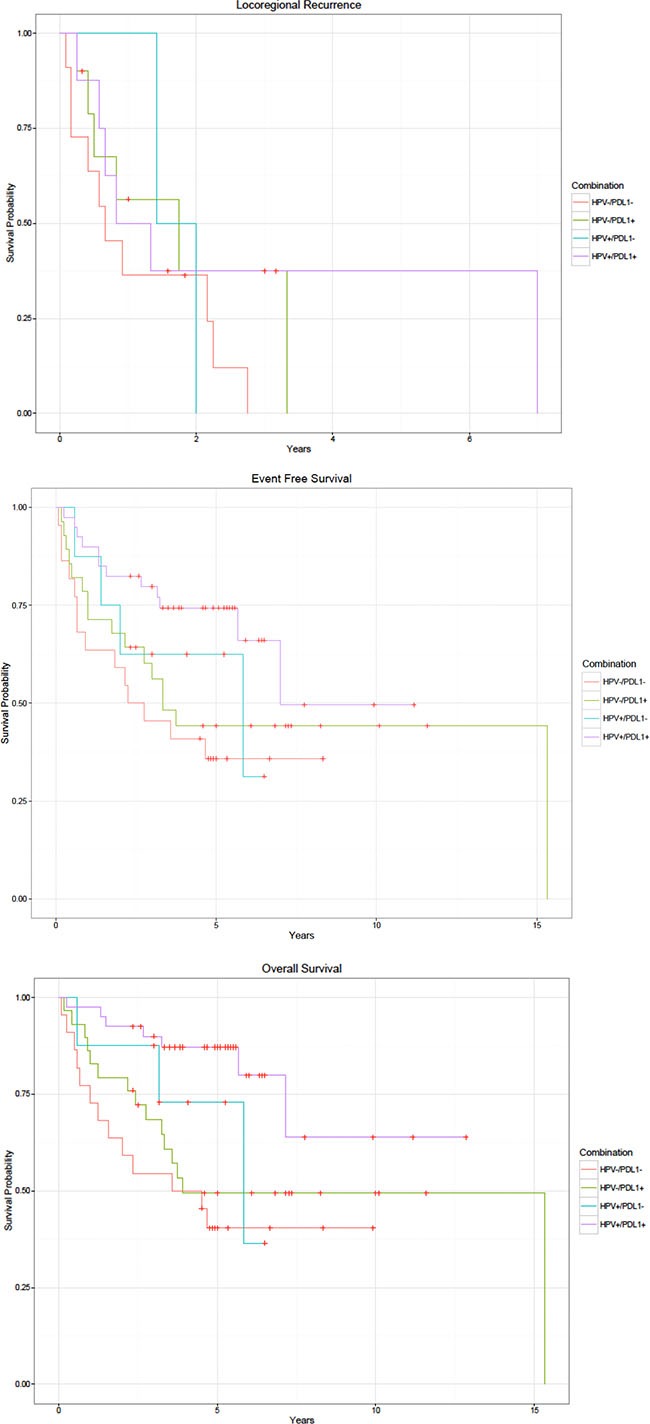
Kaplan-Meier curves by HPV and PD-L1 status (A) Locoregional recurrence. (B) Event free survival. (C) Overall survival.

Effects of a combination of HPV and PD-L1 on outcomes are shown in multivariate Kaplan-Meier models (Figure 1A–1C). There was no significant difference in the risk of locoregional recurrence by HPV/PD-L1 combination (*p* = 0.548). The best event-free survival was observed in HPV-positive/PD-L1-positive cancer and the worst survival was seen in HPV-negative/PD-L1-negative cancer (*p* = 0.05). Similarly, patients with HPV-positive/PD-L1-positive cancer had the best overall survival compared with patients with HPV-negative/PD-L1-negative cancer (*p* = 0.006). Relative to those patients with HPV-negative/PD-L1-negative disease who had the highest risk of death, patients with HPV-positive/PD-L1-positive cancers had a 2.85 fold lower risk of developing an event (HR 0.35, 95% CI: 0.16–0.79) and a 4.5 fold lower risk of death (HR = 0.22, 95% CI: 0.09–0.53) (Table 5). There was no significant difference in the risk of locoregional recurrence by HPV/PD-L1 combination (*p* = 0.548).

**Table 5:** Association between HPV status and PD-L1 status on outcome after adjusting for clinical variables

HPV and PD-L1 status	Time to LR failure HR (95% CI)	Time to event HR (95% CI)	Time to death HR (95% CI)
HPV+/PD-L1+ (*n* = 40)	0.54 (0.19, 1.50)	0.35 (0.16, 0.79)	0.22 (0.09, 0.53)
HPV+/PD-L1− (*n* = 8)	0.75 (0.15,3.79)	0.65 (0.19, 2.23)	0.56 (0.14, 2.23)
HPV-/PD-L1+ (*n* = 28)	0.58 (0.20, 1.62)	0.70 (0.29, 1.65)	0.67 (0.26, 1.72)
HPV-/PD-L1− (*n* = 22)	1	1	1

## DISCUSSION

The aim of this study was to analyze the expression of PD-L1 in a large series of tonsillar cancer and to evaluate its clinical relevance. We focused our study on patients with carcinoma of the tonsil, the subsite of oropharyngeal cancer with the highest HPV positivity rate, to limit the variation in prognosis among tumors involving different subsites of the oropharynx [1, 2]. We found a strong correlation between HPV positivity and PD-L1 expression (83.3% vs 56.9%, *p* < 0.05). Patients with PD-L1-positive cancer were more likely to be never smokers and non-drinkers. However, there was no statistically significant difference in PD-L1 positivity by smoking status within the HPV-positive group, probably due to the small number of cases. A similar study of 133 OSCC cases using a non-commercial PD-L1 antibody did not show any significant difference in PD-L1 expression by HPV status. [14] However, they used 20% membrane staining as the positive cut-off point instead of the ≥ 1–5% staining commonly reported in the literature and the > 1% used in our study [15].

We found statistical evidence that gender and HPV status were important prognostic factors in patients with tonsillar cancer. This is consistent with many prior studies showing the better prognosis of HPV-positive OSCC compared to HPV-negative smoking-related OSCC [3, 16, 17]. However, PD-L1 status alone was not prognostic for outcome after adjusting for other known prognostic factors. Importantly, we identified a significant interaction between HPV status and PD-L1 status in terms of risk of an event and overall survival. The best outcome was seen in patients with both HPV-positive and PD-L1-positive tumors. Patients with HPV-negative/PD-L1-negative tumors had a 2.85 fold increased risk of developing an event and 4.5 fold increased risk of death from any cause compared to patients with HPV-positive/PD-L1-positive tumors, after adjusting for clinical variables.

Since the US Food and Drug Administration approved ipilimumab for treatment of advanced melanoma in 2011, there has been an immense interest in immunotherapy as a cancer treatment. Immunotherapies with checkpoint blockade antibodies that block CTLA-4 and PD-1 (or its ligand PD-L1) can restore and augment cytotoxic T-cell responses against cancers, leading to durable responses and prolonged overall survival with tolerable toxicity. PD-L1 is the most commonly used biomarker in immunotherapy. Upregulation of PD-L1 expression detected by immunohistochemistry has been reported in many different cancer types, e.g. melanoma (40–100%), non-small cell lung cancer (35–95%), and multiple myeloma (93%). A recent meta-analysis demonstrated that PD-L1 expression is significantly associated with response to anti-PD-1/PD-L1 antibodies in patients with non-small cell lung cancer.[15] However, a proportion of apparently PD-L1-negative patients also benefits from anti-PD-1 therapy. Therefore, expression of PD-L1 is not a perfect predictive biomarker and, in our view, should not be used as a marker for selection for treatment with anti-PD-1/PD-L1 antibodies. This view is also shared by a recent editorial on the value of PD-L1 as a prognostic marker in head and neck cancer [12]. The predictive value of PD-L1 could be weakened by the availability of different antibodies and variation in cut-off values used in the earlier studies. However, there are now several commercially available PD-L1 immunohistochemistry antibodies and a direct comparison of three of these in 500 cases showed a very high correlation of 91–95% [18]. Another potential predictor of response to immunotherapy is PD-L2. The majority of studies evaluating PD-L2 expression did not find a significant correlation between survival and PD-L2 expression [19–21].

Mutation load has been shown to correlate with response to anti-PD1 immunotherapy [22]. Rizvi et al. performed whole exome sequencing of non-small cell lung cancers treated with pembrolizumab and showed that higher mutation load correlated with better response rate (63% vs 0%), more durable clinical benefit (73% vs 13%), and better progression-free survival (14.5 vs 3.7 months)[23]. Moreover, efficacy of pembrolizumab also correlated with the molecular smoking signature, higher neoantigen burden and DNA repair pathway mutations. However, further studies are needed to determine the correlation between PD-L1 expression, mutation burden and response to immunotherapy. In our study, smoking-related OSCC had a significantly lower PD-L1 expression. The Cancer Genomic Atlas Network performed a comprehensive profile of 279 head and neck cancers including 22 cases of OSCC [24]. They reported a distinct pattern of somatic genomic alteration in HPV-related and smoking-related cancers. HPV-related cancers were characterized by mutation of the PIK3CA gene, novel alterations involving loss of TRAF3 function, and amplification of the cell cycle gene E2F1. Smoking-related cancers demonstrated loss-of-function p53 mutations and CDKN2A inactivation. We previously showed that HPV-positive OSCC were significantly less likely to have a p53 mutation than HPV-negative OSCC (25.8% vs 46.7%, *p* = 0.0021) [25]. The underlying difference in mutation load between HPV-positive and HPV-negative OSCC may account for the difference in PD-L1 expression.

Cancers that are densely infiltrated by lymphocytes are considered to reflect host immune response against malignancy [26]. Emerging evidence suggests that the degree of T-cell infiltration of primary tumors consistently predicts favourable outcomes in a number of malignancies, including head and neck cancer. The Head and Neck SPORE Program investigators suggested that higher TIL levels were associated with better relapse free survival and overall survival [27]. Although there was no difference in the absolute presence or absence of TILs by HPV status, we found that HPV-positive tonsillar cancers were more likely to have a greater TIL infiltration.

In conclusion, our study showed that the pattern of PD-L1 expression in tonsillar cancer is related to HPV and smoking. There was a strong interaction between HPV status and PD-L1 status with the worst outcome seen in patients with HPV-negative/PD-L1-negative cancer. The results of this study will help to guide future clinical trial design in immunotherapy based on PD-L1 expression in tonsillar cancer.

## MATERIALS AND METHODS

### Study population

The study cohort comprised 99 patients with tonsillar squamous cell cancer treated with curative intent. The study was approved by the ethics committee of Sydney Local Area Health Service (Protocol X12–0141). Demographic and clinicopathological data were obtained from the institutional database. Selection criteria included availability of primary tumor material, availability of baseline clinicopathological data and follow up data. Patients were followed up for the occurrence of an event, defined as recurrence in any form or death from any cause, for a median of 56 months after diagnosis. The study pathologists reviewed the histology and tumor grade in all cases. Cancers were staged using the American Joint Committee in Cancer Staging System 7^th^ edition. No patient was treated with immunotherapy.

### Laboratory studies

#### HPV testing

Evidence that HPV is transcriptionally active is needed to establish oncogenicity in OSCCs. Overexpression of p16 resulting from downregulation of retinoblastoma protein by HPV E7 oncoprotein has been used as a surrogate marker of HPV expression in previous studies.[7, 28] An HPV-positive cancer was defined as one testing positive for HPV DNA and with p16 overexpression on immunohistochemistry.[7, 29] In our study, the presence and type of HPV DNA were determined by E6-based multiplex tandem PCR assay using a modification of the Tandem method of Stanley and Szewezuk.[30] This assay can simultaneously detect and identify 21 HPV types (16, 18, 31, 33, 35, 39, 45, 51, 52, 56, 58, 59, 66, 68, 70, 73, 82, 53, 6, 11 and 26). Measured quantities of equine herpesvirus were introduced during the extraction process to monitor the efficiency of DNA extraction and removal of PCR inhibitors. The expression of p16 was determined by semiquantitative immunohistochemistry using the JC2 p16 antibody (Neomarkers, Fremont, USA) as previously reported.[31] Staining was typically strong and diffuse across the nucleus and cytoplasm of cancer cells and recorded as positive if seen in more than 50% of cancer cells.[32]

### PD-L1 immunohistochemistry /Tumor-Infiltrating Lymphocytes (TIL)

Immunohistochemical staining for PD-L1 (Cell Signaling clone E13LN run at 1:200 concentration) was conducted on an Autostainer Plus (Dako - Agilent Technologies) using 4μm-thick tissue sections. Sections were dehydrated for 1 hour at 60°C and heat-induced epitope retrieval was performed using EnVision FLEX target retrieval solution for 20 minutes at 97°C. The sections were then cooled to room temperature in TBST Wash buffer for 5 minutes. PD-L1 staining underwent signal amplification using the Envision flex Mouse linker (K8022) followed by Envision FLEX kit (K8023) with a DAB chromagen (Dako – Agilent technologies) prior to counterstaining with hematoxylin. Scoring of PD-L1 staining was determined as the percentage of tumor cells showing positive membrane staining and any sample displaying any unequivocal tumor staining, however focal, was deemed to be PD-L1 positive.

The percentage of the tumor containing infiltrating lymphocytes was assessed semi-quantitatively using a four-tier scale: 0 = 0%, 1 = < 25%, 2 = 25− < 75%, 3 = > 75%. All scoring was conducted blinded to HPV status and clinical outcomes.

### Statistical analyses

Comparisons between demographic and clinicopathological characteristics were undertaken using *t*-tests for continuous variables and chi-squared tests for categorical variables. The Kaplan-Meier method was used to construct time-to-event curves. Locoregional failure was defined as clinical, radiological and/or pathological evidence of recurrence at the primary site or in the regional nodal area. Times to locoregional failure, any event and death from OSCC or death from any cause were calculated from the date of diagnosis. Patients were censored at last follow-up / distant recurrence / death where applicable, or excluded if they had incomplete information on recurrence. For the analysis of time to death from any cause, patients were censored at last follow-up if they were alive. Univariate and multivariable time-to-event analyses were performed using Cox proportional hazards regression modelling.
